# Comments on: Segmental Uterine Wall Resection for Placenta Accreta Spectrum Disorder: For what Purpose?

**DOI:** 10.1055/s-0038-1675199

**Published:** 2018-10

**Authors:** Shigeki Matsubara

**Affiliations:** 1 Department of Obstetrics and Gynecology, Jichi Medical University, Tochigi, Japan

Dear Editor,

I read with great interest the article “Conservative surgical treatment of a case of placenta accreta” by Biyik et al.^1^ Although they used the term placenta accreta syndrome to illustrate the condition, the International Federation of Gynecology and Obstetrics (FIGO) recently recommended the use of placenta accreta spectrum (PAS) disorders, which are subclassified into creta, increta, and percreta. While creta is an adhesion abnormality in which the placenta adheres to, but does not invade, the myometrium, increta and percreta are invasion abnormalities, with the placenta invading into and beyond the myometrium, respectively.^2^ The terminology of PAS disorder avoids ambiguity, especially regarding the dual meaning of accreta, and I wish to use it in this discussion. Biyik et al^1^ partially resected the uterus, with the placenta attaching to it, and then reconstructed it: they performed uterine segmental resection for percreta. It is important to point out that at the time of this procedure, tubal ligation was also performed. My concern regards the combination of segmental resection with tubal ligation.

Fundamentally, there are four strategies for treating PAS disorder: i) intentional placental removal followed by uterine reconstruction, ii) segmental resection of the uterus, iii) placenta left in situ approach, and iv) hysterectomy (cesarean or intentionally delayed).^3^ ^4^ As Biyik et al stated, the gold standard treatment strategy for PAS disorder is cesarean hysterectomy.^4^ However, recent advances in various hemostatic procedures and the accumulation of technical developments have facilitated i) or ii), depending on the situation.^5^ The strategy of iii) may also become an option in some selected cases.^3^ The most important point is: while i), ii), and iii) fundamentally aim for future fertility, iv) does not.^3^ ^4^

In the case described by Biyik et al^1^, “due to the preoperative approval, tubal ligation was performed.” Tubal reconstruction or in-vitro fertilization are two options for such patients, if they wish to have another baby, and, thus, fertility still remains, in a strict meaning. However, in this case, this was the patient's fourth baby and the context indicates that she did not desire a fifth one. I understand that retaining the uterus, irrespective of fertility, may preserve the sexual identity and improve a woman's quality of life, in some cases. However, Biyik et al^1^ made no mention of whether this was the reason for preserving the uterus in this patient.

It was fortunate that she bled only ∼ 1,000 mL during segmental resection. However, since this patient did not have placenta previa, with the PAS disorder confined to the anterior uterine wall, it is expected that cesarean hysterectomy may have not been very difficult. Hysterectomy for PAS disorder with placenta previa is difficult because the pelvis is almost completely occupied by the swollen lower uterine segment,^4^ which is referred to as “snowman sign”.^6^

There are no definite data on which option causes less bleeding and which causes less adverse events, cesarean hysterectomy or segmental resection.^3^ Biyik et al^1^ may have considered that segmental resection may cause less bleeding and less adverse events than hysterectomy in this particular patient, irrespective of the attempt to preserve fertility. However, this was not stated.

Any procedure may be good if it provides a good outcome. Thus, I commend Biyik et al^1^ for this attempt. However, why was cesarean hysterectomy, a gold standard treatment, not employed in this particular patient, even though she did not wish to preserve fertility? How a surgery is performed is one thing, and why it was done is another. If a procedure other than the gold standards is chosen, there should be a concrete reason and indication for it.

## Notes

Authors' Reply
**Comment 1**
Although they used the term placenta accreta syndrome to illustrate the condition, the International Federation of Gynecology and Obstetrics (FIGO) recently recommended the use of placenta accreta spectrum (PAS) disorders, which are subclassified into creta, increta, and percreta.
**Answer to Comment 1**
We are very pleased with your interest and thank you for your comment. Placenta accreta spectrum disorders are named differently in different sources. Placenta accreta disorder is named different in the literature. FIGO recommended the use of placenta accreta spectrum (PAS) disorders, which are subclassified into accreta, increta, and percreta.^7^ However, placenta accreta syndrome is used in a few other sources.^8^ ^9^

**Comment 2**
However, this was her fourth baby and the context indicates that she did not desire a fifth one. I understand that retaining the uterus, irrespective of fertility, may preserve the sexual identity and improve a woman's quality of life, in some cases. However, Biyik et al made no mention of whether this was the reason for preserving the uterus in this patient. Any procedure may be good if it provides a good outcome. Thus, I commend Biyik et al for this attempt. However, why was cesarean hysterectomy, a gold standard treatment, not employed in this particular patient, even though she did not wish to preserve fertility? How a surgery is performed is one thing, and why it was done is another. If a procedure other than the gold standards is chosen, there should be a concrete reason and indication for it.
**Answer to Comment 2**
Fertility-preserving surgical methods also reduce the impact on a woman's societal status and self-esteem associated with the loss of her uterus. Despite the fact that the patient was not expecting fertility preservation and did want tubal ligation, the patient's husband wanted preserving of the uterus, if possible, during the intraoperative period. To reduce the impact on a woman's societal status and self-esteem associated with the loss of her uterus, we opted for conservative surgery.^7^ In this case, the adhesive portion of the placenta was close to the Kehr incision line, so it was suitable for segmental uterine wall resection. We want to say ‘since it had been determined that there was no excessive bleeding, you could make a choice and thus opted for conservative surgery.
